# Adenosquamous carcinoma coexisting with intraductal papillary mucinous neoplasm of the pancreas: a case report

**DOI:** 10.1186/s13256-023-03798-0

**Published:** 2023-03-02

**Authors:** Hirozumi Sawai, Yuka Kiriyama, Hiromasa Kuzuya, Yoshiaki Fujii, Shuhei Ueno, Shuji Koide, Masaaki Kurimoto, Kenji Yamao, Yoichi Matsuo, Mamoru Morimoto, Hajime Koide, Atsushi Kamiya

**Affiliations:** 1 Department of Surgery, Narita Memorial Hospital, Hanei-Honmachi 134, Toyohashi, Aichi 4418029 Japan; 2 Department of Pathology, Narita Memorial Hospital, Toyohashi, Aichi Japan; 3 Department of Gastroenterology, Narita Memorial Hospital, Toyohashi, Aichi Japan; 4 Department of Gastroenterological Surgery, Nagoya City University Graduate School of Medical Sciences, Kawasumi 1, Mizuho-Cho, Mizuho-Ku, Nagoya, Aichi 467-8601 Japan

**Keywords:** Adenosquamous carcinoma, Intraductal papillary mucinous neoplasm, Pancreas, *KRAS*, *GNAS*

## Abstract

**Background:**

Adenosquamous carcinoma of the pancreas is a rare variant, with a worse prognosis than pancreatic ductal adenocarcinoma; moreover, it has characteristic clinical and histopathological features. Studies have mentioned the differentiation of intraductal papillary mucinous neoplasms into mucinous/tubular adenocarcinomas; however, their transdifferentiation into adenosquamous carcinoma remains unclear.

**Case presentation:**

An 80-year-old Japanese woman was referred to our hospital for further examination of multiple pancreatic cysts. Enhanced computed tomography after close follow-up for 6 years revealed a new nodule with poor enhancement on the pancreatic body. Distal pancreatectomy and splenectomy were performed. Histopathological examination revealed an adenosquamous carcinoma with coexisting intraductal papillary mucinous neoplasms; moreover, the intraductal papillary mucinous neoplasms lacked continuity with the adenosquamous carcinoma. Immunohistochemical analysis revealed squamous cell carcinoma and differentiation from adenocarcinoma to squamous cell carcinoma. Gene mutation analysis revealed *KRAS*^G12D^ and *KRAS*^G12R^ mutations in adenosquamous carcinoma components and intraductal papillary mucinous neoplasm lesions, respectively, with none showing the mutation of *GNAS* codon 201. The final histopathological diagnosis was adenosquamous carcinoma with coexisting intraductal papillary mucinous neoplasms of the pancreas.

**Conclusions:**

This is the rare case of adenosquamous carcinoma with coexisting intraductal papillary mucinous neoplasms of the pancreas. To investigate the underlying transdifferentiation pathway of intraductal papillary mucinous neoplasms into this rare subtype of pancreatic cancer, we explored gene mutation differences as a clinicopathological parameter.

## Background

Pancreatic cancer is a severe disease, and its incidence and mortality rates are on the rise [1]. Despite improvements in multidisciplinary treatment to improve the prognosis of this advanced disease, there remain suboptimal treatment outcomes due to the rapid local tumor spread or metastatic dissemination. There are several histological subtypes of pancreatic cancer. Among them, adenosquamous carcinoma (ASC) is a rare variant that accounts for only 1–4% of all pancreatic neoplasms [2, 3]. Pancreatic ASC has a worse prognosis than pancreatic ductal adenocarcinoma (PDA); moreover, it has some unique clinical and histopathological characteristics [3, 4].

Intraductal papillary mucinous neoplasms (IPMNs) are classified into subtypes based on their histological and imaging characteristics [5]. Most cases of main duct IPMNs are of the intestinal type; additionally, the large and complex form of intestinal-type IPMNs can have invasive carcinoma, typically of the mucinous type, with relatively lazy behavior [6]. On the other hand, gastric-type IPMNs are typically low grade, with only a small proportion developing into carcinoma. However, gastric-type IPMNs behave like conventional PDA upon development into carcinomas, typically of the tubular type [7, 8]. Although it has been reported that IPMN develops into mucinous carcinoma and tubular adenocarcinoma, transdifferentiation of IPMN into ASC remains unclear.

This article describes a rare case of an ASC with coexisting IPMNs of the pancreas. Further, we examined mutations in driver genes for progression in ASC and IPMNs to investigate the possibility of malignant transdifferentiation in pancreatic neoplasm.

## Case presentation

**Fig. 1:**
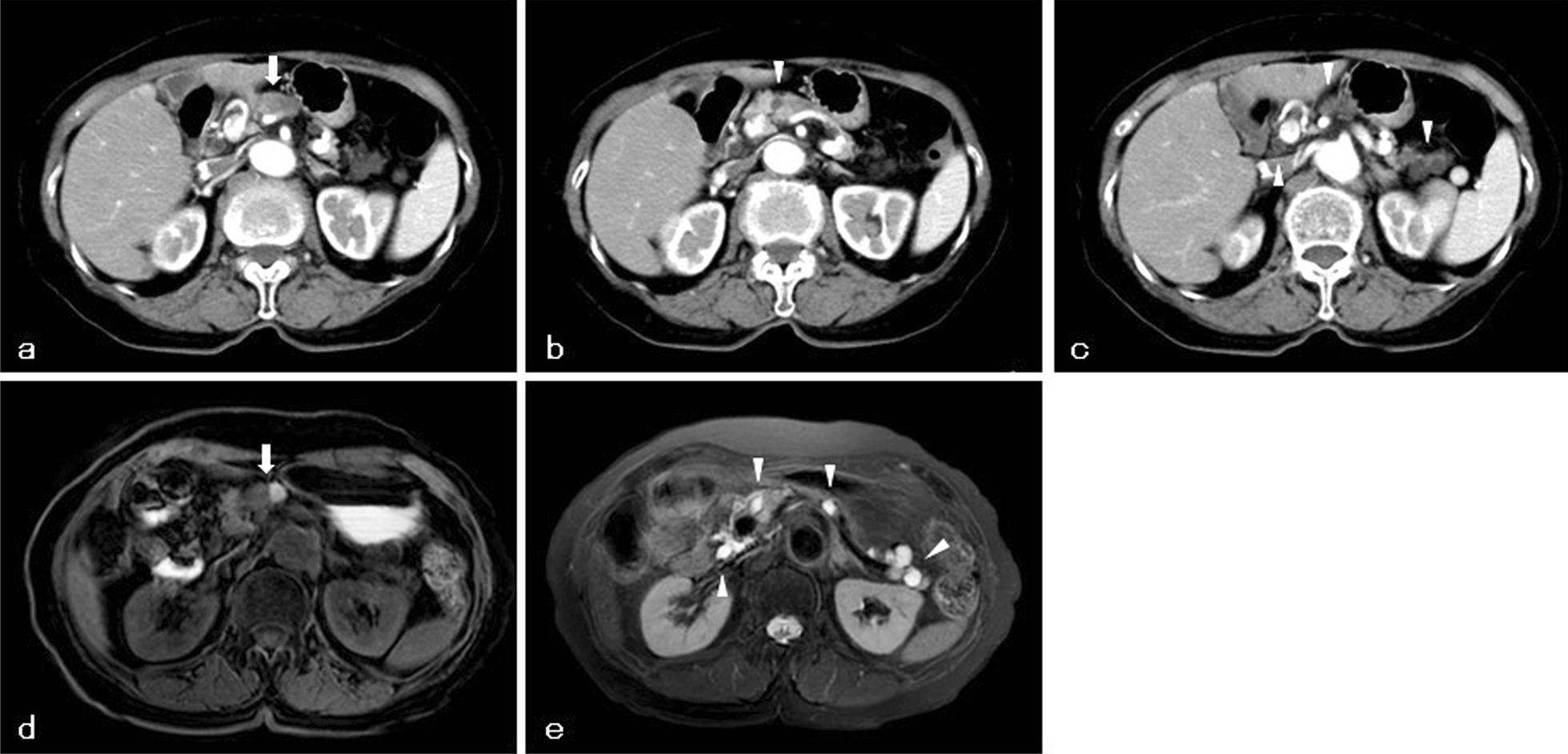
Enhanced computed tomography and magnetic resonance imaging images. Enhanced computed tomography revealed a poorly enhanced nodule (white arrow) on the pancreatic body (**a**) and a cystic lesion (white arrowhead) adjacent to the tumor (**b**). Additionally, several cystic lesions (white arrowheads) were detected throughout the pancreas (**c**). Magnetic resonance imaging revealed a tumor with a partially high-intensity area (white arrow) on the pancreatic body (**d**). Further, several cystic lesions (white arrowheads) were detected throughout the pancreas on T2-weighted magnetic resonance imaging (**e**)

**Fig. 2:**
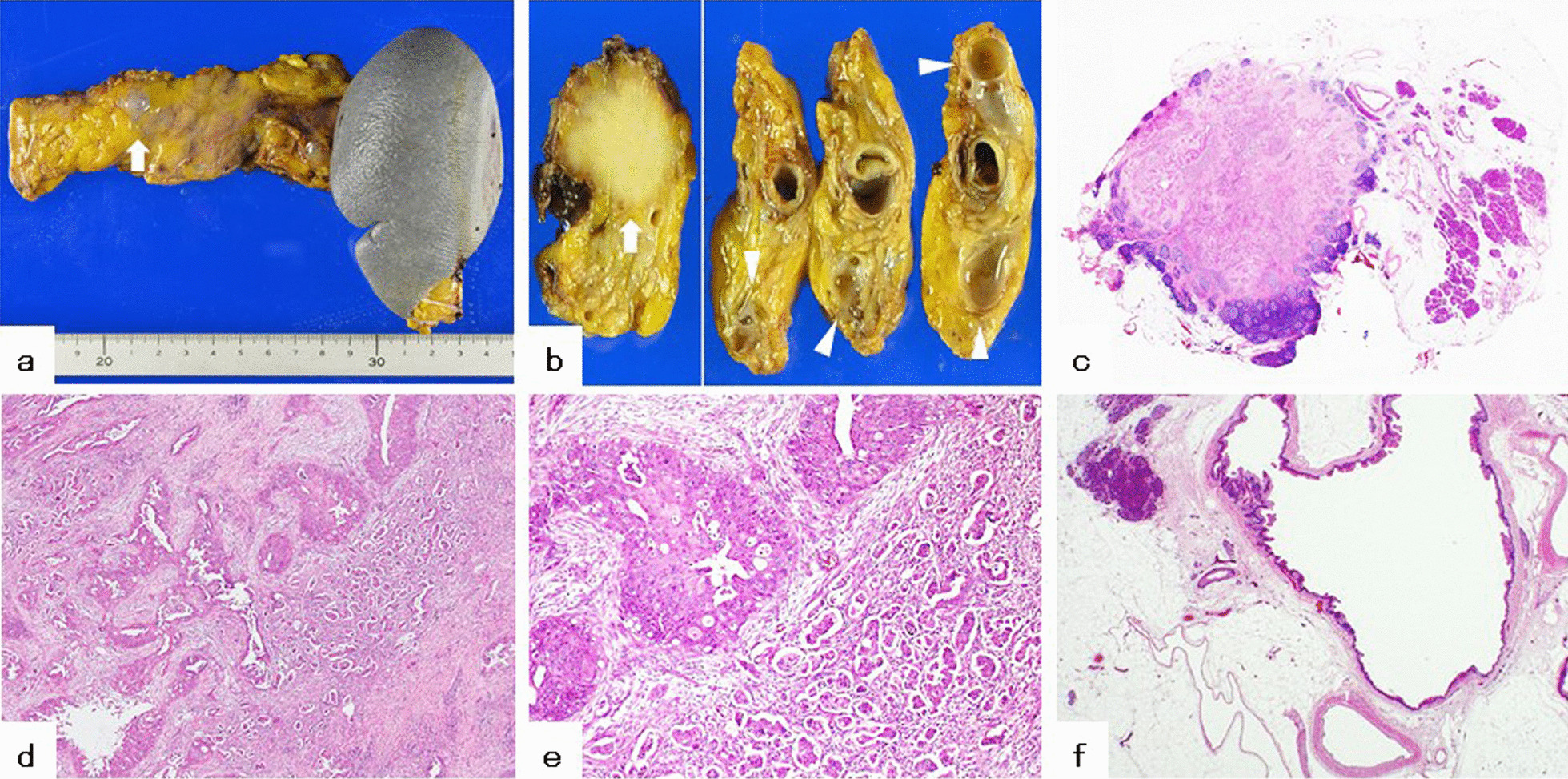
Macroscopic and microscopic pathology (hematoxylin and eosin stain) of the surgical specimen. Macroscopically, an invasive tumor with a diameter of 15 mm (**a**, **b**, white arrow) and cystic lesions (**b**, white arrowheads) were observed. There was an invasive tumor with a diameter of 15 mm and cystic lesions around the tumor (**c**; loupe magnification, ×4). The solid tumor was proliferating with irregular small and medium-sized glandular cavities with interstitial fibrosis (**d**; original magnification, ×40). There were partially invasive micropapillary patterns, and differentiation into squamous cell carcinoma was noted (**e**; original magnification, ×200). The columnar epithelium of intraductal papillary mucinous neoplasm cysts observed around the solid tumor had a papillary appearance (**f**; original magnification, ×20)

An 80-year-old Japanese woman was referred to our hospital with multiple cystic lesions throughout the pancreas identified on abdominal ultrasonography. She had no complaints associated with pancreatic cystic lesions. The lesions were up to 13 mm in size. After close follow-up for 6 years, a new nodule was found on the pancreatic body that had a diameter of 18 mm and poor enhancement on enhanced computed tomography (CT); moreover, the largest cyst grew to a diameter of 18 mm (Fig. 1a–c). The serum carbohydrate 19-9 antigen (CA19-9) level remained within the normal range (< 37 U/mL) during the 6-year follow-up period. T1-weighted magnetic resonance imaging (MRI) newly showed a partially high-intensity occupying lesion with a diameter of 15 mm on the pancreatic body (Fig. 1d). T2-weighted MRI revealed multiple high-intensity cystic lesions throughout the pancreas (Fig. 1e). There was no dilatation of the main pancreatic duct, or connection of cystic lesions to the pancreatic ductal system. Endoscopic ultrasonography (EUS) revealed a hypoechoic well-defined tumor with a diameter of 15 mm on the pancreatic body. Through EUS‐guided fine needle aspiration (FNA) biopsy, a tumor sample was obtained and histopathologically confirmed as adenocarcinoma of the pancreatic body. Further, diagnostic imaging led to a diagnosis of the multiple cystic lesions throughout the pancreas as IPMNs. The cysts had diameters of up to 18 mm; moreover, there were no findings suggestive of malignancy, including enhancing mural nodules, and thickened/enhancing cyst walls. On the basis of these results, we performed distal pancreatectomy and splenectomy with lymph node resection. Macroscopically, we observed an invasive tumor with a diameter of 15 mm and cystic lesions around the tumor (Fig. 2a, b). Microscopically, the main tumor was proliferating with irregular medium and small glandular cavities, accompanied by interstitial fibrosis (Fig. 2c, d). We partially observed invasive micropapillary pattern and differentiation into squamous cell carcinoma (Fig. 2e). Since the columnar epithelium of these cysts was papillary, the many dilated cysts observed around the main tumor were diagnosed as IPMNs (Fig. 2f). IPMNs lacked continuity with the adenocarcinoma or squamous cell carcinoma components. The margins of the resected pancreas and the six resected lymph nodes were negative for ASC. Based on the American Joint Committee on Cancer (AJCC) classification, the ASC was stage IA (T1a, N0, M0) [9].

**Fig. 3:**
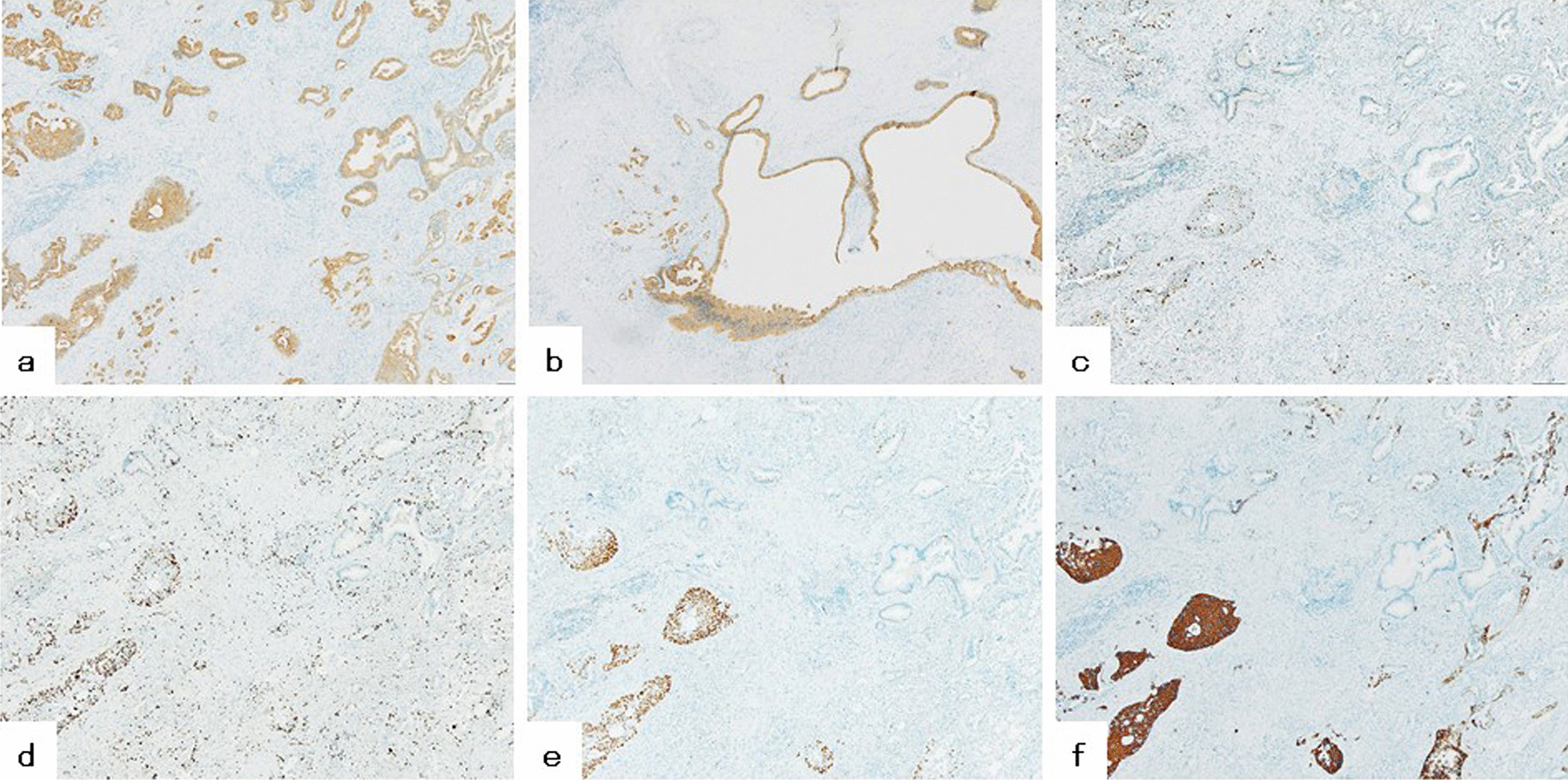
Immunohistochemical analysis of the surgical specimen (original magnification, ×40). Immunohistochemical analysis revealed MUC1 expression in both adenosquamous carcinoma (**a**) and intraductal papillary mucinous neoplasm (**b**). Expression of p53 (**c**) and Ki67 (**d**) was observed only in adenosquamous carcinoma and, specifically, on both the adenocarcinoma and squamous cell carcinoma components. p40 expression was detected only in the squamous cell carcinoma components of adenosquamous carcinoma (**e**). There was stronger CK5/6 expression in the squamous cell carcinoma component than in the adenocarcinoma component (**f**)

Table 1 presents the findings of immunohistochemical analysis. MUC1 was expressed by both ASC and IPMN cells (Fig. 3a, b). ASC also showed strong expression of p53 and Ki-67, but the IPMNs did not (Fig. 3c, d). Only the squamous cell carcinoma component of ASC showed p40 expression (Fig. 3e). CK5/6 was expressed in ASC cells; moreover, it was more strongly expressed in the squamous cell carcinoma component than in the adenocarcinoma component (Fig. 3f). Assessment of gene mutations revealed *KRAS*^G12D^ and *KRAS*^G12R^ mutations in ASC and IPMNs, respectively (Table 2). The mutation of *GNAS* codon 201 was not detected in either ASC or IPMNs. Based on these results, the final histopathologic diagnosis was pancreatic ASC and IPMN.

**Table 1:** Immunohistochemical findings

	ASC		IPMN
	AC components	SC components	
MUC1	+	+	+
p53	+	+	−
Ki-67	+	+	−
p40	−	+	−
CK5/6	+	+	−

*ASC* adenosquamous carcinoma, *AC* adenocarcinoma, *SC* squamous cell carcinoma, *IPMN* intraductal papillary mucinous neoplasm

**Table 2:** Gene mutations

	ASC	IPMN
*KRAS* codon 12	G12D	G12R
*GNAS* codon 201	WT	WT

*ASC* adenosquamous carcinoma, *IPMN* intraductal papillary mucinous neoplasm, *WT* wild type

Given the patient’s age, oral administration of S-1 (an oral 5-fluorouracil prodrug) was initiated twice daily at a dose of 50 mg/day for 14 consecutive days as postoperative adjuvant chemotherapy; she was also advised to rest for 7 days. No recurrence was noted after 9 months of follow-up.

## Discussion and conclusions

ASC of the pancreas is defined as a mixed tumor with ductal adenocarcinoma component, and ≥ 30% malignant squamous cell carcinoma component [10]. The prognosis of pancreatic ASC is worse than that of PDA; furthermore, the median survival following ASC is only 5.6–13.0 months [4, 11–14]. The mechanisms underlying ASC development remain unclear; however, several theories have been proposed regarding histopathogenesis. The first theory suggests the existence of pancreatic cancer stem cells [15]. The second theory, which is also known as collision theory, suggests that two histologically distinct tumors develop and integrate independently [3, 16]. Recent studies have suggested the existence of ASC progenitor cells in some cancers, given the genetic and immunohistochemical similarities coexisting in tubular and squamous ASC components [17–20]. Based on these theories and reports, the monoclonal pathway could be a logical hypothesis for ASC development. The existence of this monoclonal pathway must include a gene that promotes squamous differentiation of the adenocarcinoma from the progenitor cells [21].

There has been increasing incidence of pancreatic IPMN, given the recent advances in diagnostic imaging. Patients with IPMN have a favorable postoperative prognosis; recent studies have reported a 5-year postoperative survival rate for patients with IPMN of 89–97.5% [22–24]. It was demonstrated that IPMNs differentiated into adenocarcinoma in 47% and 17% of patients with the main pancreatic duct type and branch type as precancerous lesions, respectively; it was also demonstrated that it usually takes > 10 years to develop into a malignant invasive tumor [25]. Omori *et al*. identified three different pathways (sequential, branch-off, and *de novo* subtypes) through which IPMNs progress to PDAs by performing detailed histologic and genetic analysis of PDAs and concurrent IPMNs [26]. Additionally, they reported differences in the genes that were mutated, especially *KRAS* and *GNAS*, between PDA arising from IPMNs and *de novo* PDA.

Close follow-up of our patient was undertaken on the basis of the International Consensus Guidelines 2012 for the Management of IPMN and MCN of the Pancreas [5]. In our patient, the poorly enhanced nodule, ASC, could not be confirmed on CT at the first visit of the patient, which was 6 years ago, nor could it be confirmed on the CT taken a year prior to the surgery. On the other hand, the cystic lesion with a diameter of 13 mm that was identified on CT 6 years ago had grown in size to 18 mm at the time of surgery. This clinical course of this patient indicates that the pancreas, which can be the origin of IPMNs, may develop *de novo* malignant tumors regardless of whether IPMNs differentiate into cancer or not. Therefore, there is a need for regular and close follow-up by diagnostic imaging for patients with IPMNs.

Adjuvant chemotherapy significantly improved disease-free survival, compared with surgery alone, in patients undergoing gross curative resection of pancreatic cancer [27, 28]. As previously mentioned, the prognosis of pancreatic ASC is worse than that of PDA. Although this case was stage IA according to AJCC classification, postoperative adjuvant chemotherapy was performed for the purpose of improving the postoperative prognosis and according to the patient’s strong desire.

*KRAS* gene is localized to chromosome 12q12.1 and is a proto-oncogene that belongs to the RAS family. It encodes KRAS, which is stimulated by upstream pathways, including growth factors, to promote the action of other genes and acts in the signaling transduction pathway of EGFR. KRAS regulates proteins involved in cell survival, angiogenesis, proliferation, and metastasis. A point mutation in *RAS* (*KRAS*/*NRAS*) could be associated with various cancers, especially pancreatic and colorectal cancer. Recent studies have confirmed that over 90% of patients with early-stage pancreatic intraepithelial neoplasia (PanIN), which is the most common precursor lesions, express mutant *KRAS* [29, 30].

*GNAS* gene is present at chromosome 20q13.32 and is also known as the *GNAS* complex locus, which is composed of 13 exons and 12 introns. *GNAS* encodes the stimulatory guanine nucleotide-binding protein (G protein) α subunit (Gαs), and the product of the *GNAS* gene acts as the mediator of the G-protein-coupled receptor signaling pathway [31]. *GNAS* was identified as a driver gene for IPMN in 2011 [32, 33]. *KRAS* and *GNAS* mutations are often and rarely, respectively, detected in PDA. Contrastingly, *GNAS* mutation is considered an IPMN-specific gene abnormality; therefore, PDA with *GNAS* mutation is considered as having differentiated from IPMN lesions [26, 32–34].

Differentiation from IPMN to ASC is extremely rare, with three reports of ASC arising from IPMN present in the literature thus far [35–37]. Furthermore, to our knowledge, there has been no report that demonstrates IPMN lacking continuity with the ASC components. In our case, immunostaining results for CK5/6, which is a marker of squamous cell carcinoma, suggested the probability of differentiation from adenocarcinoma to squamous cell carcinoma. Moreover, there were different *KRAS* mutations in ASC and IPMN components. In contrast with *KRAS* mutation, *GNAS* mutation was not detected in either ASC or IPMN components. Certainly, ASC could be a carcinoma developed in a multifocal IPMN with an ASC phenotype. Alternatively, our results of gene mutation analysis also suggest that ASC in this case may have been derived from PanIN.

In summary, we present a rare case of coexisting ASC and IPMN of the pancreas. Our findings suggest that differences in gene mutations may facilitate the exploration of the IPMN malignant transformations and the origins of pancreatic cancer. The biological characteristics of ASC remain unclear; therefore, there is a need for further research to determine the factors for differentiation to ASC.

## Data Availability

The data supporting the findings of this case report are available within the article.

## Abbreviations

ASC: Adenosquamous carcinoma
IPMN: Intraductal papillary mucinous neoplasm
PDA: Pancreatic ductal adenocarcinoma
CT: Computed tomography
CA19-9: Carbohydrate 19-9 antigen
MRI: Magnetic resonance imaging
EUS: Endoscopic ultrasonography
FNA: Fine needle aspiration
AJCC: American Joint Committee on Cancer
PanIN: Pancreatic intraepithelial neoplasia
